# Philopatry drives genetic differentiation in an island archipelago: comparative population genetics of Galapagos Nazca boobies (*Sula granti*) and great frigatebirds (*Fregata minor*)

**DOI:** 10.1002/ece3.386

**Published:** 2012-10-04

**Authors:** Iris I Levin, Patricia G Parker

**Affiliations:** 1 Department of Biology, University of Missouri – St. Louis, One University Blvd.St. Louis, Missouri, 63121; 2 Whitney R. Harris World Ecology Center, University of Missouri – St. Louis, One University Blvd.St. Louis, Missouri, 63121; 3 WildCare Center, Saint Louis Zoo, One Government Dr.St. Louis, Missouri, 63110

**Keywords:** Galapagos, natal philopatry, population genetics, seabird

## Abstract

Seabirds are considered highly mobile, able to fly great distances with few apparent barriers to dispersal. However, it is often the case that seabird populations exhibit strong population genetic structure despite their potential vagility. Here we show that Galapagos Nazca booby (*Sula granti*) populations are substantially differentiated, even within the small geographic scale of this archipelago. On the other hand, Galapagos great frigatebird (*Fregata minor*) populations do not show any genetic structure. We characterized the genetic differentiation by sampling five colonies of both species in the Galapagos archipelago and analyzing eight microsatellite loci and three mitochondrial genes. Using an *F*-statistic approach on the multilocus data, we found significant differentiation between nearly all island pairs of Nazca booby populations and a Bayesian clustering analysis provided support for three distinct genetic clusters. Mitochondrial DNA showed less differentiation of Nazca booby colonies; only Nazca boobies from the island of Darwin were significantly differentiated from individuals throughout the rest of the archipelago. Great frigatebird populations showed little to no evidence for genetic differentiation at the same scale. Only two island pairs (Darwin – Wolf, N. Seymour – Wolf) were significantly differentiated using the multilocus data, and only two island pairs had statistically significant φ_ST_ values (N. Seymour – Darwin, N. Seymour – Wolf) according to the mitochondrial data. There was no significant pattern of isolation by distance for either species calculated using both markers. Seven of the ten Nazca booby migration rates calculated between island pairs were in the south or southeast to north or northwest direction. The population differentiation found among Galapagos Nazca booby colonies, but not great frigatebird colonies, is most likely due to differences in natal and breeding philopatry.

## Introduction

Island archipelagos have played an important role in our understanding of diversification and speciation. Despite low species diversity, the Galapagos Islands have an exceptionally large proportion of endemic species across flora and fauna (Snell et al. 2002), and these species have supported a substantial body of research on the processes related to inter-island or inter-population variation and differentiation. The Galapagos are located on the equator, approximately 1000 km off the coast of South America and have never been connected to the mainland. The isolation of the archipelago, and the defining features of island systems (restricted land mass, clearly defined geographic boundaries) make for a useful system in which to understand how populations are shaped by the evolutionary forces of genetic drift, mutation, and selection. Due to their restricted area, islands typically harbor smaller populations than are found on continents, which can lead to a stronger effect of genetic drift. The differentiation resulting from genetic drift can be countered by any homogenization caused by gene flow, common in highly mobile organisms that migrate from their natal sites. Galapagos organisms exhibit high variation with respect to population differentiation: on one end of the spectrum, Galapagos penguins (*Spheniscus mendiculus*) (Nims et al. 2008) and Galapagos doves (*Zenaida galapagoensis*) (Santiago-Alarcon et al. 2006) have high levels of gene flow between island populations, while land iguanas (*Conolophus* sp.)(Tzika et al. 2008), Galapagos hawks (*Buteo galapagoensis*) (Bollmer et al. 2005), and Galapagos flightless cormorants (*Phalacrocorax harrisi*) (Duffie et al. 2009) show high levels of differentiation between island populations.

Within seabirds, one finds an apparent paradox between mobility and philopatry; seabirds are some of the most vagile organisms (e.g., Dearborn et al. 2003; Weimerskirch et al. 2006), and yet birds of some seabird species do not disperse and instead breed in their natal colonies (e.g., Huyvaert and Anderson 2004). Seabirds presumably encounter few geographic barriers to dispersal (at least within ocean basins), but indirect (genetic) evidence suggests that population differentiation can be strong in many species (Friesen et al. 2007). The Galapagos Islands support large numbers of seabirds, including those with large distributions (e.g., great frigatebird [*Fregata minor*], blue-footed booby [*Sula nebouxii*], red-footed booby [*S. sula*], magnificent frigatebird [*F. magnificens*]), as well as endemic species (e.g., Galapagos petrel [*Pterodroma phaeopygia*], flightless cormorant). The great frigatebird breeds throughout the Pacific, the South Atlantic, and the Indian Oceans. The Nazca booby (*S. granti*) is a common, resident Galapagos seabird throughout the archipelago that was elevated to species status in 2002 after morphological (Pitman and Jehl 1998) and genetic (Friesen et al. 2002) evaluation demonstrated marked differences from individuals belonging to other Pacific subspecies. The Nazca booby has a more restricted range than its sister species, the masked booby (*S. dactylatra*), with breeding colonies located primarily on oceanic islands on the Nazca tectonic plate, namely the Revillagigedo Islands in Mexico, Clipperton and Malepo Islands in Colombia, the Galapagos Islands and La Plata Islands in Ecuador (Pitman and Jehl 1998), and records from the Lobos de Afuera Islands, Peru (Figueroa 2004) and from Oahu and Tern Island in Hawaii (Vanderwerf et al. 2008).

We used eight variable microsatellite DNA markers and mitochondrial DNA (mtDNA) sequence data from three genes to describe the population genetic structure of Galapagos great frigatebirds and Nazca boobies. There is some indication that both sexes of great frigatebirds are somewhat natally philopatric (Metz and Schreiber 2002); however, physical distance within an island colony in the northwestern Hawaiian Islands did not correlate well with degree of genetic similarity, as one would predict given high natal philopatry (Cohen and Dearborn 2004). Breeding and natal dispersal of Nazca boobies is extremely limited (Huyvaert and Anderson 2004), thus we predict that high philopatry will promote population differentiation between Galapagos Nazca booby colonies on different islands. These patterns suggest that great frigatebird populations should show less population differentiation than the Nazca booby populations. Due to high vagility of both species and documented rare long-distance dispersal events (Booby: Huyvaert and Anderson 2004; Frigatebird: Dearborn et al. 2003), we make no prediction at the scale of the Galapagos archipelago, regarding geographic distance as an isolating barrier for either species.

## Materials and Methods

### Sample collection

**Figure 1:**
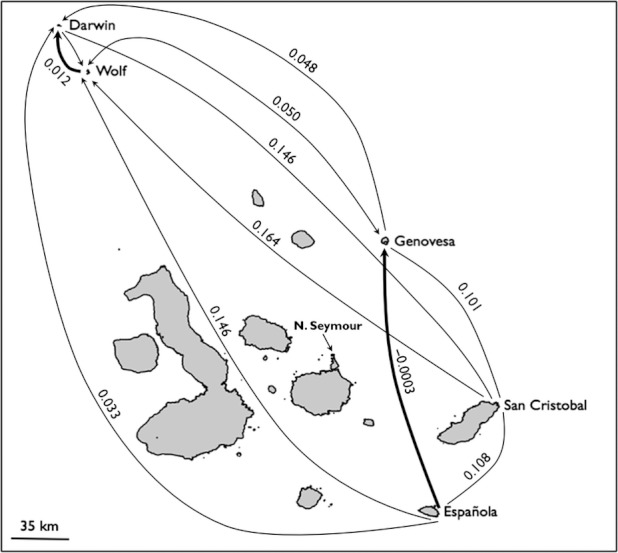
Map of the Galapagos Islands with sampled islands labeled. Numbers next to arrows are **pair-wise F_ST_** values calculated for colonies of Nazca boobies (*Sula granti*) using eight microsatellite loci. Arrows show directional migration (rates calculated in BayesAss). Thick arrows indicate higher migration rates (0.18–0.29) while thinner arrows represent lower migration rates (0.01–0.06). Lines with no arrowheads have directional migration rates <0.01. Bidirectional migration rates >0.01 are indicated by lines with arrowheads on both ends.

Seabirds were sampled in July 2007, June-July 2008, June 2010, and July 2011 from six islands in the Galapagos (Darwin, Española, Genovesa, North Seymour, San Cristobal, and Wolf, Fig. 1). Because only two Nazca boobies were captured on North Seymour, these individuals were removed from the analyses. Great frigatebirds captured on San Cristobal were not breeding at the time of sampling, so we did not include them in the analysis. Sample sizes per island can be found in Tables 1 and 2. Birds were captured by hand and two drops of blood, collected via brachial venipuncture, were preserved in 500 μL of lysis buffer (Longmire et al. 1988).

**Table 1:** Total number of alleles (*N*_a_), Nei's unbiased gene diversity (h), and rarefied allelic richness (R_S_ for each colony and locus, R_T_ for all colonies combined) for populations of Galapagos Nazca boobies (*Sula granti*). Sample size = 133; sample sizes per island: Darwin = 12, Española = 51, Genovesa = 27, San Cristobal = 29, Wolf = 14

	Darwin			Española			Genovesa			San Cristobal			Wolf			Total		
						
Locus	*N*_a_	h	R_S_	*N*_a_	h	R_S_	*N*_a_	h	R_S_	*N*_a_	h	R_S_	*N*_a_	h	R_S_	*N*_a_	h	R_T_
Sv2a-53	3	0.638	3.00	4	0.649	3.24	3	0.649	3.00	3	0.603	3.00	4	0.585	3.86	5	0.727	3.4
Sn2b-83	4	0.649	4.00	6	0.640	4.68	5	0.720	4.68	4	0.662	3.89	4	0.704	4.00	7	0.737	4.6
Sn2a-123	2	0.391	2.00	2	0.503	2.00	2	0.492	2.00	2	0.506	2.00	2	0.519	2.00	2	0.501	2.0
Sv2a-47	2	0.228	2.00	2	0.318	2.00	2	0.372	2.00	2	0.373	2.00	2	0.138	1.98	2	0.316	2.0
Ss2b-110	2	0.083	2.00	3	0.148	2.23	3	0.352	2.93	3	0.222	2.60	2	0.071	1.86	3	0.194	2.6
Ss2b-48	3	0.518	3.00	4	0.646	3.23	3	0.570	2.95	3	0.612	3.00	3	0.553	2.86	4	0.602	3.2
RM4-D07	4	0.772	4.00	7	0.698	4.80	6	0.636	4.89	4	0.552	3.22	4	0.590	3.98	7	0.704	5.0
RM4-G03	8	0.870	8.00	7	0.758	5.66	9	0.788	7.02	8	0.822	6.83	6	0.817	5.84	10	0.810	7.6
All loci	28			35			33			29			27			40		
Mean		0.519	3.50		0.545	3.48		0.572	3.68		0.544	3.32		0.497	3.30	5	0.585	3.8

**Table 2:** Total number of alleles (*N*_a_), Nei's unbiased gene diversity (h), and rarefied allelic richness (R_S_ for each colony and locus, R_T_ for all colonies combined) for populations of Galapagos great frigatebirds (*Fregata minor*). Sample size = 114; sample sizes per island: Darwin = 15, Española = 29, Genovesa = 27, North Seymour = 28, Wolf = 15

	Darwin			Española			Genovesa			North Seymour			Wolf			Total		
						
Locus	*N*_a_	h	R_S_	*N*_a_	h	R_S_	*N*_a_	h	R_S_	*N*_a_	h	R_S_	*N*_a_	h	R_S_	*N*_a_	h	R_T_
Fmin1	6	0.671	6	7	0.685	5.96	7	0.620	5.94	6	0.720	5.86	6	0.690	6	8	0.671	5.95
Fmin4	2	0.333	2	6	0.429	4.92	5	0.363	4.56	4	0.283	3.62	4	0.402	4	7	0.358	3.82
Fmin11	5	0.452	5	3	0.448	2.96	3	0.402	2.99	3	0.436	2.95	4	0.562	4	5	0.451	3.58
Fmin6	5	0.679	5	8	0.771	7.23	8	0.813	7.26	8	0.800	7.41	7	0.798	7	9	0.787	6.78
Fmin18	7	0.790	7	6	0.735	5.28	8	0.822	6.99	7	0.759	5.53	7	0.771	2	8	0.775	5.36
Fmin8	2	0.400	2	2	0.491	2.0	2	0.503	2.0	2	0.420	2.0	2	0.457	7	2	0.492	2.00
Fmin10	10	0.881	10	10	0.792	8.22	8	0.793	7.45	10	0.818	8.44	7	0.821	10	11	0.810	8.82
Fmin2	10	0.890	10	13	0.909	11.1	13	0.907	11.0	14	0.892	10.9	14	0.926	14	17	0.899	11.4
All loci	54			55			54			54			51			67		
Mean		0.637	5.88		0.658	5.96		0.653	6.02		0.641	5.84		0.678	6.75	8.4	0.656	6.09

### Laboratory analyses

DNA was extracted following a standard phenol-chloroform extraction protocol (Sambrook et al. 1989). DNA concentrations were estimated by spectrophotometry and diluted to approximately 20 ng/μL for subsequent genetic analyses. Microsatellite markers developed specifically for great frigatebirds were used for this species (Table 2) (Dearborn et al. 2008). Microsatellite primers specific for Nazca boobies were not available. Therefore, we used a number of published markers developed for related booby species that showed sufficient levels of polymorphism (Table 1) (Faircloth et al. 2009; Morris-Pocock et al. 2010a; Taylor et al. 2010a). Twenty-five primer pairs were tested, and 17 were rejected due to monomorphism or poor amplification. Aside from three of the frigatebird primers which were fluorescently labeled (Fmin3, Fmin6, Fmin8), one of the primers in each set (typically the shorter one) had a 5′ CAG tag applied (Glenn and Schable 2005). We added a “pigtail” (GTTT) to the 5′ end of the primer lacking the CAG tag to facilitate the addition of adenosine by the taq polymerase (Brownstein et al. 1996). Details on PCR protocol and fragment analysis can be found in the supplemental information. Genemapper v.4.01 (Applied Biosystems, Life Technologies, Carlsbad, California) software was used to analyze the fragment analysis results. All individual genotypes were manually scored, 10% of the total samples were re-amplified and genotyped from the original template DNA across all loci, and roughly one-third of all homozygotes were re-run to ensure we were not incorrectly assigning genotypes due to allelic dropout.

We amplified fragments of three mitochondrial genes, cytochrome *b* (cyt *b*) (780 bp) and NADH dehydrogenase subunit 2 (ND2) (566 bp), and cytochrome oxidase I (COI) (700–800 bp) for all great frigatebirds and a subset of the Nazca boobies (*n* = 50). The subsample of Nazca boobies was selected at random, but checked to ensure equal numbers of each sex. Primers for cyt *b* were B3 and B6 (T. Birt, unpubl. data; Morris-Pocock et al. 2010b), ND2Metl (O. Haddrath, unpubl. data; Hailer et al. 2011) and H5766 (Sorenson et al. 1999) were used to amplify ND2, and the entire COI gene was amplified using L6615 and H8121 (Folmer et al. 1994) followed by sequencing with internal primers socoiF1 (Chaves et al. 2008 modified from Herbert et al. 2003) and H6035COI_Tyr (Chaves et al. 2008). Details for the PCR reactions, template cleanup, and sequencing can be found in the supplemental information. DNA sequences were obtained using an Applied Biosystems 3100 DNA analyzer at the University of Missouri – St. Louis using BigDye Terminator v3.1 Cycle Sequencing chemistry.

### Population genetic structure analyses

#### Microsatellite DNA analysis

Deviation from Hardy–Weinberg equilibrium (HWE) was tested for each locus with allele randomizations within populations (1000 permutations) and over all populations (10,000 permutations) in FSTAT v. 2.9.3.2 (Goutdet 2001). Genetic variation for each locus within each population was quantified using number of alleles and genetic diversity (Nei 1972) in FSTAT and HP-RARE (Kalinowski 2005) was used to calculate rarefied allelic richness per site-locus combination. We tested for the presence of null alleles using ML-NullFreq (Kalinowski, http://www.montana.edu/kalinowski/Software/MLNullFreq.htm). Deviations from linkage equilibria were tested in Arlequin v.3.5.1.2 (Excoffier and Lischer 2010) using ln likelihood ratio G-tests. Arlequin was used to estimate pairwise differentiation, F_ST_ (Weir and Cockerham 1984), between all colony pairs. R_ST_ (Slatkin 1995), a similar estimate that allows for a stepwise mutation model was calculated for all colony pairs in FSTAT. A hierarchical Analysis of Molecular Variance (AMOVA) was run in Arlequin if some population differentiation was found. For the Nazca boobies, we ran the AMOVAs testing for structure using three groups (Darwin + Wolf; Genovesa + Española; San Cristobal) and two groups (Darwin + Wolf + Genovesa + Española and San Cristobal).

Genotype clustering was evaluated using a Bayesian method implemented in STRUCTURE v.2.3.3 (Pritchard et al. 2000). The most probable number of populations, *k*, was determined using the second order rate of change in posterior probabilities between runs of different k as described in Evanno et al. (2005). We performed three runs per *k* (*k* = 1 through *k* = 8) using the *locprior* setting, the admixture model, correlated allele frequencies, and a burn-in of 200,000 cycles followed by 500,000 additional cycles. We also performed shorter runs using different settings (no-admixture model, runs without the *locprior* setting) to evaluate the importance of model choice. Results were averaged for the runs and the program DISTRUCT v.1.1 (Rosenberg 2004) was used to construct a visual output from STRUCTURE using the number of populations with the highest likelihood.

Migration rates were estimated using BayesAss v.1.3 (Wilson and Rannala 2003), which evaluates gene flow using a model that does not assume migration-drift equilibrium. Default values were used: 3,000,000 Markov chain Monte Carlo iterations, 1,000,000 burn-in iterations, sampling every 2000 iterations, and initial values of delta for allele frequencies, migration rates and inbreeding set at 0.15. We tested for a relationship between geographic distance and genetic differentiation (isolation by distance) using a Mantel test implemented in the program IBD v.1.52 (Bohonak 2002) on log-transformed geographic distances and Slatkin's linearized F_ST_ values. Geographic distances between colonies were calculated using Google Earth. Recent population bottlenecks were tested for using the software BOTTLENECK v1.2.02 (Cornuet and Luikart 1997). BOTTLENECK detects recent bottleneck events by comparison of allelic diversity and heterozygosity. Allelic diversity decays faster than the correlated measure of diversity, heterozygosity, after a population has experienced a recent reduction, and therefore, heterozygosity excess can be used to infer recent bottlenecks. BOTTLENECK was run using the parameters for the Infinite Allele Model (Maruyama and Fuerst 1985) and sign tests were used to determine statistical significance.

#### Mitochondrial DNA analyses

Mitochondrial sequences were assembled and manually checked for quality in Seqman 4.0 (DNASTAR, Madison, Wisconsin) and aligned using BioEdit v.7.0.9.0 (Hall 1999). The mitochondrial dataset, containing segments of ND2, cytochrome *b* and COI was tested for neutrality using Tajima's D (Tajima 1989) tests implemented in DnaSP v.5.10.01 (Librado and Rozas 2009). Standard diversity indices (haplotype and nucleotide diversity) were calculated in DnaSP. φ_ST_ values for all pair-wise colony comparisons were calculated in Arlequin and median joining haplotype networks were calculated in Arlequin and constructed in HapStar (Teacher and Griffiths 2011). Unique mitochondrial haplotypes can be found in GenBank (accession numbers JX569150-JX569187).

## Results

### Diversity within populations

All eight microsatellite loci for both species were found to be in Hardy–Weinberg equilibrium for all populations and no loci showed any signature of null alleles. Overall, we detected 40 alleles in 133 Nazca boobies (Table 1) and 67 alleles in 114 great frigatebirds (Table 2). Allele numbers per locus in Nazca boobies varied from two to 10 (mean = 5) and from two to seventeen (mean = 8.75) in great frigatebirds. Seven private alleles were found in Nazca booby populations, three from the San Cristobal population, three from the Española population and one from the Genovesa population. Ten private alleles were found in great frigatebirds, five from the Genovesa population, three from the Española population, and one each from both Darwin and Wolf. Genetic diversity, measured as number of alleles (N_a_), Nei's unbiased genetic diversity (h), and rarefied allelic richness (R_s_) varied between different populations (Table 3 for *S. granti*, Table 4 for *F. minor*).

**Table 3:** Pair-wise F_ST_ values for Nazca boobies (*Sula granti*) from microsatellites (*n* = 133) above the diagonal and pair-wise φ_ST_ values from mtDNA (*n* = 50) below the diagonal

	Darwin	Española	Genovesa	San Cristobal	Wolf
Darwin		0.033*	0.048*	0.146*	0.012
Española	0.239*		−0.0003	0.108*	0.049*
Genovesa	0.263*	0.070		0.101*	0.050*
San Cristobal	0.302*	−0.019	−0.042		0.164*
Wolf	0.184*	0.032	0.042	0.080	

* Denotes F_ST_ and φ_ST_ values with *P*-values < 0.01.

**Table 4:** Pair-wise F_ST_ values for great frigatebirds (*Fregata minor*) from microsatellites (*n* = 114) above the diagonal and pair-wise φ_ST_ values from mtDNA (*n* = 108) below the diagonal

	Darwin	Española	Genovesa	North Seymour	Wolf
Darwin		0.004	0.017	−0.004	0.040*
Española	0.039		−0.002	−0.006	0.009
Genovesa	0.034	−0.028		0.010	0.007
North Seymour	0.111*	0.018	0.010		0.027*
Wolf	−0.018	0.002	0.002	0.059*	

* Denotes F_ST_ and φ_ST_ values with *P*-values < 0.01.

In the Nazca booby populations genetic diversity, h, ranged from 0.071 to 0.870, with a mean of 0.58 and rarefied allelic richness, R_s_, ranged from two to eight (mean = 3.8). Average genetic diversity per population was more uniform, ranging from 0.497 in Wolf to 0.572 in Genovesa. Recent population bottlenecks were detected in three of the five colonies: Española, Genovesa, and San Cristobal. In all three cases, seven of the eight loci showed a relative heterozygosity excess and *P*-values for the sign tests were 0.042, 0.048, and 0.040 for Española, Genovesa, and San Cristobal, respectively.

In the great frigatebird populations, genetic diversity ranged from 0.283 to 0.926, with a mean of 0.656. Rarefied allelic richness ranged from 2 to 14 (mean = 6.02) and average genetic diversity per population was even, ranging from 0.64 in the North Seymour and Darwin populations to 0.68 in the Wolf population. No recent bottlenecks were detected in great frigatebird populations.

**Figure 2:**
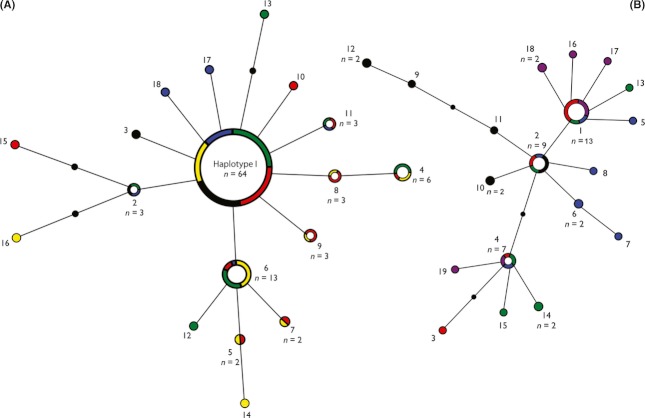
Haplotype network for Nazca boobies (*Sula granti*) (A) and great frigatebirds (*Fregata minor*) (B) based on three mitochondrial genes. Circles are proportional to the number of individuals that share the haplotypes and the colors correspond to different islands. Black = Darwin, blue = Wolf, green = Genovesa, red = Española, purple = San Cristobal (Nazca booby only), yellow = N. Seymour (great frigatebird only).

A total of 19 mitochondrial haplotypes were detected in Nazca booby samples using 2145 bp of mitochondrial DNA sequenced from 50 individuals. Overall haplotype diversity was 0.886 ± 0.028 and overall nucleotide diversity was 0.001 ± 0.0001. Haplotype and nucleotide diversity per population were very similar, and are shown in Table S1 in the supplementary data and the haplotype network is shown in Figure 2. Tests of neutrality indicated that these DNA regions are evolving in a neutral or nearly neutral fashion (Tajima's D = −1.0, *P* > 0.05). Eighteen haplotypes were identified in great frigatebirds, using 1954 bp of mitochondrial sequence from 108 individuals. Haplotype diversity was 0.644 ± 0.051 while nucleotide diversity was 0.00054 ± 0.00048. Haplotype and nucleotide diversity per population and the mitochondrial haplotype network can be found in Table S1 and Figure 2, respectively. The Tajima's D test gave no indication of non-neutrality (D = −1.64, *P* > 0.05).

### Differentiation between populations

Using microsatellite loci, we estimated global F_ST_ and R_ST_ for Nazca booby populations to be 0.070 and 0.071, respectively. Due to the similarity of values given by both F_ST_ and R_ST_, we will only report and discuss F_ST_ values for all subsequent comparisons. Eight of the 10 pair-wise estimates of structure between colonies using microsatellites were statistically significant (*P* < 0.01) (Table 3). The only colony pair comparisons that did not show significant differentiation using this approach were Darwin and Wolf (F_ST_ = 0.012), and Española and Genovesa (F_ST_ = −0.0003). In the subsample of mtDNA sequences, the global φ_ST_ was 0.127 and four of the 10 pair-wise comparisons between colonies were statistically significant (Table 3). All four significant pair-wise comparisons were between Darwin and all other colonies.

In contrast, the global F_ST_ for great frigatebird populations was 0.007. Only two of the 10 pair-wise comparisons between island colonies (North Seymour – Wolf, Darwin– Wolf) were statistically significant (Table 4), while most of the comparisons indicated high levels of gene flow between the population pairs. The mitochondrial dataset also showed weak to no genetic structure with a global φ_ST_ of 0.023 and only two significantly differentiated population pairs (North Seymour – Darwin, North Seymour – Wolf).

We found no evidence for isolation by distance in the great frigatebird populations using either dataset (multilocus data Mantel test: *r*^2^ = −0.466, *P* = 0.98; mitochondrial data Mantel test: *r*^2^ = 0.396, *P* = 0.01). In fact, according to the multilocus data, there was weak evidence for higher differentiation between closer colony pairs, driven by the significant differentiation between Darwin and Wolf, which are separated by 38 km. For Nazca boobies, there was no relationship between F_ST_ and geographic distance using multilocus data (Mantel test: *r*^2^ = 0.082, *P* = 0.07). We did, however, detect a significant positive relationship between geographic distance and φ_ST_ values for the mitochondrial data set, but the relationship explained only a very small amount of the variance and is likely driven by the significant differentiation between Darwin, a peripheral island, and all other colonies (Mantel test: *r*^2^ = 0.144, *P* = 0.02).

Hierarchical AMOVAs were run only on the Nazca booby dataset where genetic structure was detected. The AMOVA run on multilocus data showed strong support for two genetic groups (San Cristobal and all other islands) with 9.52% of the variance among groups and 2.3% of the variance among populations within groups (AMOVA, *P* = <0.001). When an AMOVA was run with three defined groups (Darwin + Wolf; Genovesa + Española; San Cristobal), there was marginal support for this structure (AMOVA; *P* = 0.06). Under this scenario, 8.77% of the variance was among groups and 0.15% was among populations within groups and 3.02% among individuals within populations.

**Figure 3:**
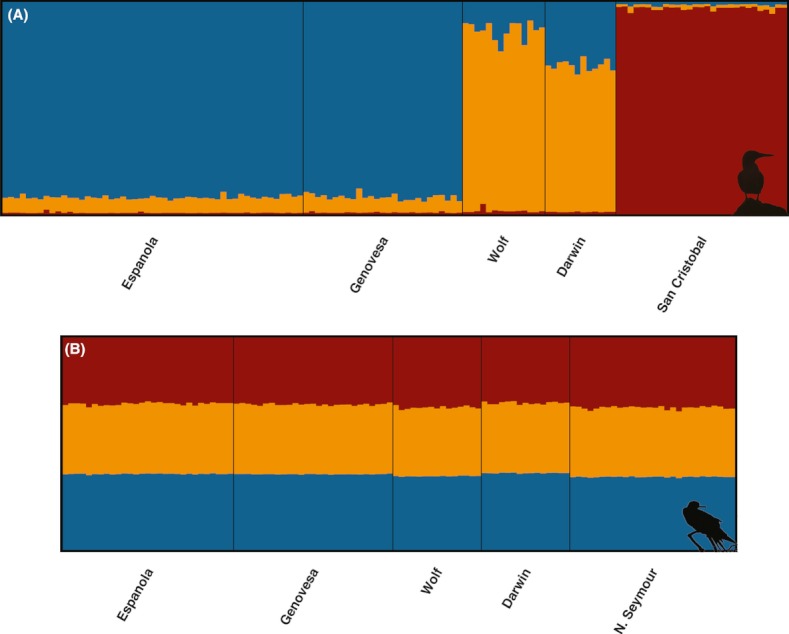
(A) Posterior probability of assignment for 133 Nazca boobies (*Sula granti*) to three genetic clusters based on a Bayesian analysis run in STRUCTURE (using *Locprior*) of variation at eight microsatellite loci. Individuals are grouped by population and the different genetic clusters are indicated by the different colors. Every vertical bar corresponds to an individual bird and the section of each bar represented by a color is equal to the number of times (calculated as a proportion of the total STRUCTURE runs) that individual was assigned to each genetic cluster. (B) Posterior probability of assignment for 114 great frigatebirds (*Fregata minor*) showing no population differentiation using the *Locprior* setting in STRUCTURE. While *k* = 3 is shown here for comparison with the results from the Nazca booby, no structure was found from *k* = 2 to *k* = 8; one genetic cluster was most likely in all cases.

The Bayesian clustering analysis performed in STRUCTURE revealed no genetic subdivision in great frigatebird populations with or without the *locprior* setting (Fig. 3, showing *locprior* results). In the case of Nazca booby populations, the analysis run with and without *locprior* indicated that three genetic clusters were most likely (Fig. 3, showing *locprior* results). One population consisted of Nazca boobies sampled from the isolated, northwestern islands of Darwin and Wolf, another included the birds from Española and Genovesa, and the third population consisted of the birds from San Cristobal (Fig. 3).

Migration rates for Nazca boobies calculated in BayesAss had a mean of 0.037 ± 0.072 SD between all pairs of island comparisons (Table 5). Rates ranged from 0.0029 (95% CI: 2.74e^−7^, 00.0182) in the case of movement from Darwin to San Cristobal to 0.2912 (95% CI: 0.2249 – 0.3265) from Española to Genovesa (Fig. 1, Table 5). Although the highest migration rate was calculated between Española to Genovesa, the migration rate in the reverse direction, from Genovesa to Española, is among the lowest calculated for Nazca boobies (0.006 ± 0.011). Seven of the 10 migration rates were either in the southeast to northwest direction or south to north direction, with only two north to south or northwest to southeast migration rates >0.01 (from Darwin to Wolf and from Wolf to Genovesa) (Fig. 1). Migration rates calculated for great frigatebirds had a mean of 0.042 ± 0.035 (Table 6). Rates ranged from 0.0065 (95% CI: 1.58e^−5^, 0.031) in the case of movement from Darwin to North Seymour to 0.2963 (95% CI: 0.2493, 0.3263) from Española to North Seymour. Migration rates of great frigatebirds did not have any clear directional pattern.

**Table 5:** Migration rates, calculated using eight microsatellite loci in BayesAss, for Nazca boobies (*Sula granti*) between colonies on five Galapagos islands. Mean and standard deviation are reported

From	To	Darwin	Española	Genovesa	San Cristobal	Wolf
↓	→					
Darwin			0.004 ± 0.007	0.010 ± 0.012	0.003 ± 0.005	0.049 ± 0.087
Española		0.056 ± 0.056		0.291 ± 0.026	0.003 ± 0.006	0.033 ± 0.043
Genovesa		0.020 ± 0.024	0.006 ± 0.011		0.003 ± 0.005	0.014 ± 0.022
San Cristobal		0.014 ± 0.018	0.003 ± 0.005	0.007 ± 0.009		0.009 ± 0.013
Wolf		0.180 ± 0.097	0.007 ± 0.011	0.011 ± 0.015	0.003 ± 0.006	

**Table 6:** Migration rates, calculated using eight microsatellite loci in BayesAss, for great frigatebirds (*Fregata minor*) between colonies on five Galapagos islands. Mean and standard deviation are reported

From	To	Darwin	Española	Genovesa	N. Seymour	Wolf
↓	→					
Darwin			0.006 ± 0.009	0.008 ± 0.010	0.007 ± 0.010	0.013 ± 0.017
Española		0.014 ± 0.019		0.009 ± 0.011	0.018 ± 0.026	0.017 ± 0.021
Genovesa		0.013 ± 0.017	0.009 ± 0.011		0.012 ± 0.018	0.023 ± 0.026
N. Seymour		0.274 ± 0.019	0.296 ± 0.021	0.290 ± 0.024		0.253 ± 0.041
Wolf		0.012 ± 0.017	0.007 ± 0.010	0.007 ± 0.010	0.009 ± 0.013	

## Discussion

Our analyses reveal that despite short geographic distances between several of the breeding colonies of Nazca boobies, there is substantial genetic differentiation within the Galapagos archipelago and that three genetically distinct populations occur within the archipelago, based on the Bayesian clustering analysis. In contrast, very weak to no population genetic structure was found in the great frigatebird using both mitochondrial and nuclear markers and we found evidence of migration of individuals between multiple colony pairs. Most of the migration rates calculated for Nazca boobies were low, with the exception of high levels of nearly unidirectional gene flow were detected between two Nazca booby colonies, Española and Genovesa. We found that several of the larger migration rates (large relative to the general trend of low numbers of individuals moving between most colony pairs) were from Española to other colonies, indicating that it might be a source population. The pronounced genetic differentiation in Galapagos Nazca boobies detected here corroborates previous mark-recapture studies that demonstrated very limited natal and breeding dispersal of Galapagos Nazca boobies (Huyvaert and Anderson 2004).

### Diversity within populations

Genetic diversity estimates within each population and across all populations were reasonably high and even for both species across populations. Our estimate of 58% (Nazca booby) and 65% (great frigatebird) heterozygosity is similar to values reported for other Galapagos taxa such as the Galapagos dove (56–65%) (Santiago-Alarcon et al. 2006) and the flightless cormorant (51–66%) (Duffie et al. 2009) and higher than Galapagos penguins (44%) (Nims et al. 2008) and Galapagos mockingbirds (*Mimus* spp.) (35%) (Hoeck et al. 2010). The caveat when comparing genetic diversity calculated from microsatellites between studies is that ascertainment bias can result from investigators selecting for polymorphic loci during primer development (Ellegren et al. 1995). Additionally, when microsatellites are used for species other than the one they were designed for (as is our case for Nazca boobies but not great frigatebirds), this ascertainment bias can lead to artificial differences due to lower polymorphism in the non-focal species (Brandström and Ellegren 2008). Whenever possible, we selected markers with a medium and comparable number of alleles. Nevertheless, results should be interpreted with caution due to ascertainment bias and the fact that population size influences genetic diversity.

Evidence for recent bottlenecks was detected in the Española, Genovesa, and San Cristobal colonies of Nazca boobies. This could be due to the El Niño Southern Oscillation (ENSO) events that raise sea surface temperature, which can negatively affect marine life in Galapagos. The 1986–1987 ENSO event, while less severe than the one in 1982–1983, caused Nazca boobies to either suspend breeding or adjust the timing of their breeding cycle (Anderson 1989). These results must be interpreted with caution due to recent literature review demonstrating underestimation of bottlenecks by moment-based estimators like the one used in this analysis (Peery et al. 2012). Additionally, Peery et al. (2012) point out that the proportion of multistep mutations is often underestimated in microsatellite datasets and therefore bottleneck tests can spuriously detect bottlenecks in stable populations.

Haplotype diversities estimated from mtDNA were fairly high (h = 0.886 for Nazca boobies, 0.644 for great frigatebirds), compared with the Galapagos flycatcher (*Myiarchus magnirostris*) (h = 0.491) (Sari and Parker 2012) and comparable to the Galapagos hawk (*Buteo galapagoensis*) (h = 0.671) (Bollmer et al. 2006). Four island colonies of Nazca boobies had three or more unique mtDNA haplotypes (Darwin = 3, Genovesa = 3 San Cristobal = 4, Wolf = 4), and the most genetically distinct island was Darwin. For great frigatebirds, 64 of 108 individuals shared one common haplotype. All island populations except Darwin had at least two unique mtDNA haplotypes and there were four haplotypes that were shared between N. Seymour and Española. The star-like shape of the frigatebird haplotype network with one common haplotype and several unique haplotypes differing by one mutational step resembles that of an organism undergoing a demographic expansion. However, we did not detect a significantly negative value for Tajima's D (−1.64, close to significant but *P* > 0.05).

### Differentiation between populations

As predicted, population differentiation was more pronounced among Nazca booby populations compared with populations of great frigatebirds, most likely driven by differences in degree of natal and breeding philopatry. According to the multilocus dataset, great Frigatebirds showed very weak to no genetic structure, with the largest F_ST_, 0.0396, between Darwin and Wolf, the two islands closest in proximity. Even with the *Locprior* setting in STRUCTURE, we detected no population subdivision among great frigatebird colonies (Fig. 3, shown at *k* = 3 for comparison with the three groups detected in the Nazca booby). Interestingly, the mitochondrial genes provide evidence for weak differentiation between N. Seymour and Darwin and N. Seymour and Wolf. These discrepancies in overall pattern calculated using different markers could be due to the timescale on which the markers provide the best resolution (mtDNA most useful for a more historical perspective and microsatellite DNA best for more recent estimates of divergence) and/or any sex bias in dispersal (maternally inherited mtDNA vs. biparentally inherited nuclear microsatellites). Although we have evidence that Galapagos great frigatebirds are genetically distinct from their non-Galapagos conspecifics (F. Hailer, unpubl. data), the birds breeding within the archipelago appear to be exchanging genes at a rate that swamps any effects of philopatry. The archipelago-wide average migration rate for great frigatebirds is similar to the one calculated for Nazca boobies; however, individual rates between colonies are more variable in the frigatebird while Nazca booby migration rates are all quite low aside from substantial movement of individuals from Española to Genovesa and from Wolf to Darwin. There is historical evidence from great frigatebirds breeding in the northwestern Hawaiian Islands demonstrating that the locations of breeding colonies are dynamic and the patterns may be explained by changes in vegetation important for nesting (Cohen and Dearborn 2004). Within Galapagos, we have anecdotal evidence of similar dynamics; the breeding colony sampled on Española in 2007 was not present in 2010 and there were no indications of prior recent breeding (old nests, chicks, juveniles). Aside from lower natal and breeding philopatry, another explanatory factor could be lack of philopatry to non-breeding site. Friesen et al. (2007) found philopatry to non-breeding site to be a strong predictor of population genetic structure. We do not have good information on philopatry during the non-breeding season for this population, but it has been shown that great frigatebirds travel great distances during the non-breeding season, and therefore may contribute to lower philopatry during the non-breeding season (Weimerskirch et al. 2006). At-sea distribution may also play a role in shaping population structure. Telemetry studies of great frigatebirds in the northwestern Hawaiian Islands indicate that, while there is a lot of variation, most of the foraging trips of great frigatebirds caring for chicks are within 200 km from their nesting colony (Gilmour et al. 2012).

Nazca boobies showed pronounced genetic differentiation. As predicted, population differentiation, as measured by F_ST_ calculated with the multilocus dataset, was statistically significant between all but two Nazca booby population pairs (Genovesa-Española; Darwin-Wolf). The gene flow between Darwin and Wolf is not surprising given that they are separated by only 38 km. Gene flow between Genovesa and Española, separated by 194 km, but not between San Cristobal and either Genovesa (140 km) or Española (87 km) is a bit more puzzling. The western tip of San Cristobal is slightly east of a straight line between Española and Genovesa, but the main seabird colonies are located on the extreme northeastern tip of the island, also the most eastern point in the archipelago, with other smaller colonies along islets on the north side. Española birds dispersing in a north-northwestern direction, and therefore not passing over the colony on San Cristobal, would explain our estimates of archipelago-wide directional migration rates, and suggest that most gene flow occurs in a northern or northwestern direction. Interestingly, gene flow was also highest between Galapagos doves sampled on Española and Genovesa, although San Cristobal was omitted from the analyses due to small sample size (Santiago-Alarcon et al. 2006). Similarly, Arbogast et al. (2006) found that Galapagos mockingbirds from Española, Genovesa, and San Cristobal had very similar mtDNA despite being considered different species.

Mitochondrial φ_ST_ values for Nazca boobies showed a different pattern than F_ST_'s calculated with microsatellites. φ_ST_ values were low for most colony pairs except for Darwin and all other colonies, which showed high levels of differentiation. Again, these discrepancies are likely due to the differences in the strength of the particular marker in resolving divergence at different timescales and could also reflect sex differences in dispersal as mtDNA provides only maternally inherited information. Although genetic distinctiveness of Darwin birds was not seen in the microsatellite analysis of pair-wise differentiation, we find a similar pattern of differentiation in the extreme corners of the archipelago: Darwin is the most northern and most western of the islands while San Cristobal is the most eastern island currently above sea level. This pattern is evident in Darwin's finches (*Geospiza*, *Camarhynchus*, *Catospiza*, and *Certhidea* spp.), where peripheral populations were found to be more genetically distinct (Petren et al. 2005). However, the larger Nazca booby colonies we sampled for this study are all arguably peripheral, so it is difficult for us to provide much support for the claim that peripheral isolation is driving this pattern of population differentiation in our system. Finally, despite the fact that, depending on the molecular markers used, different colonies emerge as the most genetically distinct, there are consistencies between the mtDNA and the multilocus datasets; several of the pair-wise relationships tell the same story for both marker types (e.g., Española and Genovesa, Darwin and San Cristobal, Darwin and Española).

No strong relationship was found between geographic distance and genetic differentiation of Nazca boobies using either mtDNA or microsatellite data. A Mantel test did detect a significant isolation by distance relationship using φ_ST_, but it appeared to be an artifact of a few points, only explaining 14% of the variation in the data. A positive relationship between geographic distance and genetic differentiation was found in Galapagos passerine birds (Petren et al. 2005; Hoeck et al. 2010), Galapagos hawks (Bollmer et al. 2005), and in flightless cormorants (Duffie et al. 2009) where distance-limited dispersal is not surprising; however, it is not surprising that we do not find isolation by distance effects in a vagile seabird on such a small geographic scale.

The Bayesian clustering analysis detected three distinct populations of Galapagos Nazca boobies: San Cristobal, Genovesa and Española, and Darwin and Wolf. These results are consistent with the genetic uniqueness of San Cristobal birds (this population, along with Genovesa, had the greatest number of private alleles), and the relative isolation of Darwin and Wolf compared with any other islands in the archipelago. Migration rate estimates indicate that the highest level of gene flow occurs from Española to Genovesa and from Wolf to Darwin. Interestingly, there is negligible gene flow from Genovesa to Española. The majority of migration rate estimates >0.01 are in a north or northwestern direction, the direction of the prevailing winds.

Galapagos Nazca booby colonies are strongly genetically structured, especially when considering the small geographic scale while great frigatebirds are not. Regarding the structure detected in the Nazca booby, some *Sulidae* species show strong phylogeographic signals and/or population genetic structure (e.g., brown booby (Morris-Pocock et al. 2011); red-footed booby (Morris-Pocock et al. 2010b), while others do not (e.g., blue-footed booby (Taylor et al. 2011); Peruvian booby (Taylor et al. 2010b)). A possible explanation for the lack of structure in the blue-footed and Peruvian booby populations is their specialization to cold-water upwelling environments such as the Humboldt Current system. When ENSO events disrupt the upwelling, successful reproduction and survival could depend on movement of individuals to more suitable breeding colonies (Taylor et al. 2011). Population differentiation in the Galapagos Nazca booby and other *Sulidae* is most likely due to strong natal philopatry. Median natal dispersal distances for Española Nazca boobies were 105 m for females and 26 m for males (Huyvaert and Anderson 2004). Only one of 198 breeding dispersal distances (breeding sites between years) within the Punta Cevallos, Española colony was greater than 25 m (Huyvaert and Anderson 2004). Documented natal dispersal from Española to other Nazca booby colonies was rare, with an estimate of 1.3% of banded nestlings moving to other surveyed islands (excluding Darwin and Wolf) (Huyvaert and Anderson 2004). This value is lower than our estimated mean migration rate across the archipelago, 0.037, but that is not surprising given that mark-recapture techniques are sure to miss some natal dispersal events leading to an underestimate. Seventeen band records were reported outside of Galapagos, indicating Galapagos Nazca boobies can disperse long distances, but will only do so rarely (Huyvaert and Anderson 2004). We gain some insights into at-sea distribution from recent telemetry work on Española Nazca boobies rearing chicks (Zavalaga et al. 2012). Individuals tended to stay within 200 km from their nesting colony during single-day trips and a maximum of 329 km were recorded for longer foraging trips (Zavalaga et al. 2012). These data, and our findings, clearly illustrate what has been called “the seabird paradox” (Milot et al. 2008) where some pelagic species show strong population genetic differentiation despite being highly mobile (Friesen et al. 2007). This paradox raises important questions involving natal and breeding dispersal, benefits of philopatry and coloniality, potential barriers (physical and non-physical) to dispersal, and colony persistence that are fundamental to our understanding of evolution in seabirds.

## Data Accessibility

mtDNA sequences (GENBANK POPSET): JX569150-JX569187.

## Supporting Information

**Table S1.** Sample sizes, number of haplotypes, haplotype diversity (*h*) and nucleotide diversity (π) for ∼2000 bp of mitochondrial DNA from Galapagos great frigatebirds (*Fregata minor*) and Nazca boobies (*Sula granti*).
