# Supplementary material for: Philopatry drives genetic differentiation in an island archipelago: comparative population genetics of Galapagos Nazca boobies (Sula granti) and great frigatebirds (Fregata minor)
Source: Ecol Evol. 2012 Oct 4;2(11):2775–87. doi: 10.1002/ece3.386 (PMC3501629; doi:10.1002/ece3.386)
Supplement: Supplementary file 1 [file ece30002-2775-SD1.docx]

Levin and Parker, Population genetics of Galapagos seabirds

*Supporting information*

*Microsatellite PCR and fragment analysis*

Ten microliter PCR reactions were run using Bioline Red taq polymerase and accompanying reagents (Bioline, Tauton, MA). Reaction conditions for PCR with primers from Taylor *et al.* (2010)(Sv2a-53, Sn2b-83, Sn2a-123, Sv2a-47) and Morris-Pocock *et al.* (2010)(Ss2b-110, Ss2b-48) were: an initial denaturation at 94°C for 2 minutes, followed by 16 cycles of 94°C for 45 seconds, 60°C for 45 seconds decreasing by 0.5°C per cycle, and 72°C for 30 seconds. Twenty-one cycles of 94°C for 45 seconds, 52°C for 45 seconds and 72°C for 30 seconds followed the touchdown cycles, as well as one final extension at 72°C for 10 minutes. Reaction conditions for PCR using primers from Faircloth *et al.* (2009)(RM4-D07, RM4-G03) were: initial denaturation at 95°C for 5 minutes, followed by 20 cycles of 95°C for 20 seconds, 65°C for 30 seconds decreasing by 0.5°C per cycle and 72°C for 90 seconds. Twenty cycles of 95°C for 20 seconds, 55°C for 30 seconds and 72°C for 90 seconds followed the touchdown cycles. The protocol ended with a 10 minute final extension at 72°C. The only deviation from the aforementioned reaction chemistry was the addition of BSA to reactions using ss2b-48. Reaction conditions for primers published in Dearborn *et al.* (2008) followed the published protocol. Microsatellites were amplified separately and then combined in two multiplex reactions with a size standard, GS500(-250)LIZ (Applied Biosystems (ABI), Life Technologies, Carlsbad, CA ), to be read by the ABI 3100 Genetic Analyzer at the University of Missouri – St. Louis.

*mtDNA PCR*

Mitochondrial DNA PCR reactions (25 microliters) were performed using the following programs. PCR conditions using cyt b primers were: initial denaturation at 95°C for 3 minutes, followed by 35 cycles of 95°C for 30 seconds, 49°C for 45 seconds and 72°C for 1 minute. The program completed with a five minute final extension of 72°C. PCR conditions for ND2 were as follows: initial denaturation at 95°C for 2 minutes, followed by 45 cycles of 95°C for 40 seconds, 52°C for 40 seconds, and 72°C for 45 seconds. One 10 minute final extension at 72°C completed the program. COI PCR reactions follow Chaves *et al.* (2008) using the published 63°C annealing temperature for *S. granti* and 62°C for *F. minor*. Reactions were performed using Takara Ex taq polymerase and accompanying reagents (Takara Bio Inc., Japan). PCR products were purified using Exonuclease I (#M0289S, New England Bio Labs Inc., Ipswich, MA) and Antarctic Phosphotase (#M0293S, New England Bio Labs Inc.). Sequencing was done at the University of Missouri – St. Louis using an Applied Biosystems 3100 DNA Analyzer with BigDye Terminator v3.1 Cycle Sequencing chemistry.

**References**

Chaves AV, Clozato CL, Lacerda DR, Sari EHR, Santos FR (2008) Molecular taxonomy of Brazilian tyrant-flycatchers (Passeriformes: Tyrannidae). *Molecular Ecology Resources*, **8**, 119-1177.

Dearborn DC, Hailer F, and Fleischer RC (2008) Microsatellite primers for relatedness and population structure in great frigatebirds (Pelecaniformes: Fregatidae). *Molecular Ecology Resources*, **8**,1399-1401.

Faircloth BC, Ramos A, Drummond H, Gowaty PA (2009) Isolation and characterization of microsatellite loci from blue-footed boobies (*Sula nebouxii*). *Conservation Genetics Resources*, **1**, 159-162.

Morris-Pocock JA, Taylor SA, Sun Z, Friesen VL (2010a) Isolation and characterization of 15 microsatellite loci for red-footed (*Sula sula*), blue-footed (*S. nebouxii*) and Peruvian (*S. variegata*) boobies. In Molecular Ecology Resources Primer Development Consortium, *Molecular Ecology Resources*, **10**, 404-408.

Taylor SA, Morris-Pocock JA, Sun Z, Friesen VL (2010) Isolation and characterization of ten microsatellite loci in Blue-footed (*Sula nebouxii*) and Peruvian Boobies (*Sula variegata*) Journal of Ornithology, **151**, 525-528.

*Supplemental table*

Table S1: Sample sizes, number of haplotypes, haplotype diversity (*h*) and nucleotide diversity (π) for ~ 2000 bp of mitochondrial DNA from Galapagos great frigatebirds (*Fregata minor*) and Nazca boobies (*Sula granti*).

| **Species** | **Island** | **n** | **Haplotypes** | ***h*** | **π** |
| --- | --- | --- | --- | --- | --- |
| *Fregata minor* |  | 108 | 18 | 0.633 | 0.00054 |
|  | Darwin | 15 | 3 | 0.257 | 0.00014 |
|  | Española | 26 | 9 | 0.668 | 0.00051 |
|  | Genovesa | 27 | 7 | 0.632 | 0.00056 |
|  | N. Seymour | 26 | 10 | 0.782 | 0.00081 |
|  | Wolf | 14 | 6 | 0.604 | 0.00037 |
| *Sula granti* |  | 50 | 19 | 0.886 | 0.00010 |
|  | Darwin | 10 | 5 | 0.822 | 0.00077 |
|  | Española | 10 | 4 | 0.644 | 0.00077 |
|  | Genovesa | 10 | 6 | 0.911 | 0.00109 |
|  | San Cristobal | 10 | 6 | 0.889 | 0.00106 |
|  | Wolf | 10 | 4 | 0.933 | 0.00098 |
